# Phylogeography of the Atlantic Blue Crab *Callinectes sapidus* (Brachyura: Portunidae) in the Americas versus the Mediterranean Sea: Determining Origins and Genetic Connectivity of a Large-Scale Invasion

**DOI:** 10.3390/biology12010035

**Published:** 2022-12-24

**Authors:** Christoph D. Schubart, Temim Deli, Giorgio Mancinelli, Lucrezia Cilenti, Alberto Gil Fernández, Silvia Falco, Selina Berger

**Affiliations:** 1 Zoology and Evolutionary Biology, University of Regensburg, 93040 Regensburg, Germany; 2 Department of Biological and Environmental Sciences and Technologies—DiSTeBA, University of Salento, 73100 Lecce, Italy; 3 National Research Council (CNR), Institute of Biological Resources and Marine Biotechnologies (IRBIM), 71010 Lesina, Italy; 4 CoNISMa, Consorzio Nazionale Interuniversitario per le Scienze del Mare, 00196 Rome, Italy; 5 Instituto de Investigación para la Gestión Integrada de las Zonas Costeras (IGIC), Universitat Politècnica de València, 46730 Grao de Gandía, Spain

**Keywords:** Atlantic Ocean, invasion biology, gene flow, genetic bottleneck, founder effect, COI mitochondrial DNA

## Abstract

**Simple Summary:**

Due to its large size and importance in commercial and recreational fishery, the blue crab, *Callinectes sapidus*, has always been a well-known crab species all along the temperate and tropical American east coast. Over the past century, there have been increasing reports of this species from Africa, Asia, and Europe. However, the corresponding introduction pathways remain a reason for speculation. Its long larval development in marine plankton and tolerance towards varying salinities are prerequisites for a successful dispersal by marine currents or in ballast waters. On the other hand, being a highly valued seafood, it is conceivable that *C. sapidus* may have been intentionally released to establish breeding populations elsewhere. The species started expanding conspicuously in the east Mediterranean after the 1930s (Nile Delta, Thessaloniki Bay). On the other hand, western Mediterranean records are much more recent and regionally confined. The reconstruction of their origin is the main goal of the current study. For that purpose, the genetic composition of populations from the American native range and from the entire Mediterranean needed to be included and used for the overall comparison. It appears that only a few founding individuals are responsible for the invasion into Spanish and Italian waters, arguing in favor of a dispersal theory.

**Abstract:**

The American blue crab *Callinectes sapidus* is a particularly successful invader in estuarine ecosystems worldwide. Despite increasing awareness of its potential harm, the invasion history and underlying genetic diversity of this species within the Mediterranean Sea remain unknown. This study constitutes the first large-scale approach to study phylogeographic patterns of *C. sapidus* in Europe, facilitated by the first comparison of all currently available COI sequence data. For this investigation, 71 individuals of *C. sapidus* were newly analyzed and the entire COI gene was sequenced and used for a comparative phylogeographic analyses. For the first time, two separately used adjacent regions of this gene were combined in a single dataset. This allowed emphasizing the prevalence of three geographically defined lineages within the native range: (1) eastern North America, including the Gulf of Mexico, (2) the Caribbean, and (3) Brazil. New data from the Mediterranean reveal that non-native populations of *C. sapidus* are characterized by a conspicuously low genetic diversity (except for Turkey, where stocking took place), and that there is surprisingly low connectivity among established populations. The occurrence of strong genetic bottlenecks suggests few founder individuals. This confirms that, even under a scenario of restricted large-scale gene flow, a very limited number of invasive individuals is sufficient for a massive impact.

## 1. Introduction

Recent studies document an alarming loss of biodiversity over the past few decades, roughly one-tenth of the global wildlife over the past twenty years alone [1]. This occurs as a consequence of the deterioration of intact nature and can be traced back to direct and indirect anthropogenic influences, resulting in biophysical disturbances of natural habitats [2]. Among them, rapid climate change impacts habitat structure and causes a shift of climate zones, playing a decisive role in the loss of biodiversity [3]. In addition to habitat destruction, biological invasions represent the second main interference in natural ecosystems and their biodiversity [4,5]. Invasive species can have a devastating impact on species loss, changes in distribution, and habitat degradation [6], mediated by predation [7], competition [8], and disease transmission [9]. The Mediterranean Sea is one of the most severely affected regions in terms of the magnitude and rate of introduction of non-indigenous species [10,11,12]. The massive invasion of non-indigenous species, and their impact on the native ecosystem, raises serious concerns because the Mediterranean Sea is considered a biodiversity hotspot [13].

The immediate impact of most non-indigenous species on newly colonized ecosystems remains largely unexplored [14,15], but a widely accepted argument is that the long-term establishment of non-indigenous species has severe negative effects, as they impact biodiversity and socio-economic values, and unbalance the functioning of ecosystems [16]. Adverse effects on biodiversity are expressed in the threat to native species due to predation, competition, displacement, and hybridization. In addition, possible effects on the overall hydrology and nutrient cycling can disrupt the structure and functioning of the entire ecosystem [17]. The threat to socio-economic values manifests itself through the impairment of fisheries and aquaculture, health and sanitation, as well as infrastructure [17]. Only a few case studies suggest that population dynamics of some non-indigenous species could also show signs of perseverance, allowing small stable populations to establish alongside native species and adapt to the structure of the local ecosystem [18].

More than 70 non-indigenous decapod species have been introduced into the Mediterranean Sea, now co-existing or competing with 309 indigenous species [19,20]. Among the non-natives, the American blue crab *Callinectes sapidus* Rathbun, 1896 is considered a particularly successful invader in Mediterranean coastal ecosystems [21,22]. It is native to Atlantic waters along the coast of Canada to Argentina, including Bermuda and the Antilles [21]. Individuals of both sexes can reach a size of up to 180 mm, while its maximum lifespan varies between 2 and 6 years, depending on local conditions [23]. The species is eurythermal and euryhaline [21]. Other biological properties, including omnivorous trophic habits, high fecundity, aggressive behavior, high mobility, and large body size [21,23,24] provide the species with attributes for great potential of successful colonization of new areas. It is therefore not surprising that its distribution area has expanded from western Atlantic waters to transitional and even freshwater environments in the North Sea, the Mediterranean Sea, the Black Sea, and even Japan in the Indo-Pacific Ocean [25,26,27].

The documented colonization of European waters began in 1900 along the Atlantic coasts of France [28], which was later followed by other regional records from the Baltic Sea, the North Sea, and the European Atlantic coast down to Portugal [22]. The date of colonization of the Mediterranean Sea is controversial, since *C. sapidus* was often misidentified as one of the Indo-Pacific invasive species, i.e., *Portunus pelagicus* (Linnaeus, 1758) in 1951 or *Portunus segnis* (Forskål, 1775) [29]. The first confirmed record of *C. sapidus* in the Mediterranean Sea was in 1947 in the Aegean Sea [22]. Nowadays, a widespread distribution across the Mediterranean Sea can be confirmed [25]. A recent study by González-Ortegón et al. [30] assessed for the first time the genetic diversity within selected populations around the Strait of Gibraltar and documented a surprising composition of merely two mitochondrial haplotypes, referred to as CSWM1 and CSWM2. However, the genetic diversity and gene flow among all other regions of the Mediterranean Sea inhabited by *C. sapidus* remained undetermined.

In the present study, our aim was to find answers to the question of the potential origin and number of introduction events, the genetic diversity of introduced populations, and the intraspecific phylogeographic relationships of *C. sapidus* in the Mediterranean Sea. In order to do so DNA-sequences of the almost complete mitochondrial gene cytochrome c oxidase subunit I (COI) from *C. sapidus* specimens from the Mediterranean Sea, namely from Albania, Italy, Romania, Spain, and Turkey, were compared with samples from the entire native distribution range in the western Atlantic, including the Gulf of Mexico and the Caribbean Sea. Maximum parsimony networks and analyses of molecular variance were applied to provide insights into the ancestry, degree of genetic differentiation, and gene flow among the studied native and invasive populations. The gene flow analyses not only provide information about the genetic connectivity among Mediterranean populations but should also allow predictions of the expected rate of future spread in this region. Considering the vast genetic variability of *Callinectes sapidus* in its native area and the many possible vectors of invasion throughout the past century, our expectation was to also encounter a relatively high genetic variability within representatives of the species in the Mediterranean Sea.

## 2. Materials and Methods

**Figure 1:**
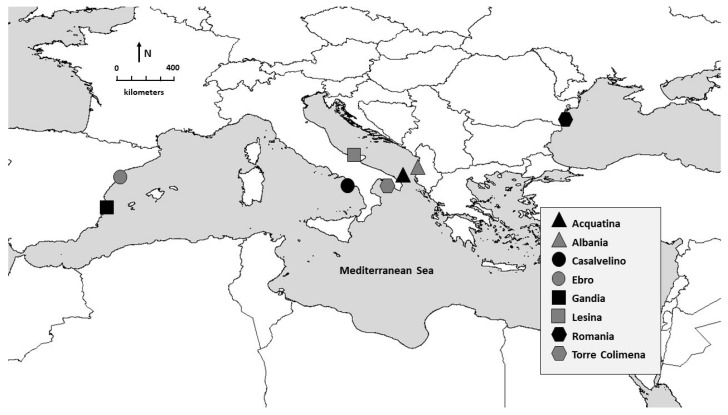
Geographical representation of sampling locations of *Callinectes sapidus* specimens used in the current study.

The here presented phylogeographic analyses are based on samples collected by us from various locations or kindly provided by colleagues. Most of the animal material originated from European waters. Sampling sites of *C. sapidus* in the Mediterranean Sea were the Italian lagoons of Acquatina, Lesina, and Torre Colimena, as well as the coastal waters of Albania, Italy, and Spain. In addition, three samples were kindly made available from the Romanian Black Sea. Four additional sequences were obtained from native populations in Brazil, Jamaica, Mexico, and Louisiana (USA). In total, new DNA sequences from 71 individuals were obtained. Table 1 lists the samples and localities, including their coordinates, and Figure 1 shows their geographic distribution.

To investigate the origin of European populations of *C. sapidus*, available sequences from GenBank (including native American and introduced Turkish populations) were also retrieved for subsequent analyses (see Tables S3–S5 in the Supplementary Material).

DNA isolation was conducted using the Puregene® kit (Gentra Systems, Minneapolis, MN, USA), after extracting muscle tissue from a pereiopod. To analyze phylogeographic relationships up to 1554 basepairs (including primers) of the mitochondrial DNA encoding the enzyme cytochrome oxidase subunit 1 (COI) of the mitochondrial respiratory chain were amplified. The PCR was performed according to the GoTAQ® Flexi DNA Polymerase Protocol (Promega, Fitchburg, WI, USA) in 25µL samples. Amplification was conducted according to the following temperature profile: initial denaturation for 4 min at 94 °C; 40 cycles of 45 s at 95 °C for denaturation, 1 min at 50 °C for primer annealing and 1 min at 72 °C for elongation; final extension for 4 min at 72 °C. The following primers were used: COL6a (5′-CAAATCATAAAGAYATTGG-3′) and COL6E (5′-ATGCAACGATGATTCTTTTCTAC-3′, new) as forward primers and COH900 (5′-ATAATTATTGCWRTYCCHAC-3′, new) and COH6 (5′-TGRTTYTTTGGHCAYCCHGAAGTHTA-3′) as reverse primers [31]. PCR products were outsourced to Macrogen Europe (Amsterdam, The Netherlands) for Sanger sequencing. Sequence files were proofread using the trace viewer software Chromas Lite 2.6.4 (Technelysium Pty Ltd. 2017, South Brisbane, QLD, AUS) and aligned with Clustal W, implemented in BioEdit 7.2.6.1 [32]. In order to visualize genealogical relationships on population level, haplotype networks were constructed with the TCS statistical parsimony algorithm, as implemented in the software Popart 1.7 [33]. In addition, maps were created with DIVA-GIS 7.5 [34] to visualize the distribution of *C. sapidus* and to provide an overview of the origin of the samples used in this study.

Quantification of overall genetic differentiation, by means of one level AMOVA, and assessment of gene flow, through pairwise comparison of genetic distances, were conducted in Arlequin 3.5.2.2 [35] for two separate datasets. The first dataset included 95 sequences of the ‘Folmer region’, corresponding to the common barcoding section of the COI gene, encompassing up to 658 basepairs (bp) exclusively from the Americas. Of these, 18 samples originated from the seagrass meadows near Pepkin in Virginia, 19 from the Rhode River in Maryland, 13 from various locations in Florida, 18 from Charleston in South Carolina, 10 from Mexico, and 17 from South America—of which 8 are from Venezuela, 4 from Colombia and 5 from Brazil (all retrieved from GenBank). The second dataset was based on 103 sequences of the so-called ‘Palumbi region’ with a length of 637 bp, restricted to sequences from Europe and North America: 20 of those samples originated from Gandía, 19 from the Ebro Delta, 10 from Casalvelino (Tyrrhenian Sea), 18 from the eastern Mediterranean and Black Sea—composed of Acquatina, Lesina, Torre Colimena, Romania and Albania (here referred to as “Eastern Basin”), whereas 18 sequences from Turkish waters and 18 from Virginia were downloaded from GenBank. Measurements corresponding to the two kinds of population genetic structure analyses were determined according to nucleotide divergence (*Φ*_ST_, based on Tajima-Nei distance) and haplotype frequencies (*F*_ST_). Significance levels of these two F-statistics were assessed by a randomization procedure with 10,000 permutations, as implemented in Arlequin [35].

Based on the outcome of the mitochondrial genealogy (see Results), the genetic makeup of the newly established populations of *Callinectes sapidus* outside the native distribution range seems to have been driven by the potential effect of a severe genetic bottleneck. In order to statistically assess this assumption, genetic diversity, demographic estimates, and population genetic differentiation were computed and compared between blue crab populations from the native and invaded sampling sites. For this purpose, the first 637 basepairs of the COI sequence data were used for a comparison of six populations from the native distribution area (Virginia, Maryland, Florida, South Carolina, Mexico, and South America) and five from newly invaded regions (Gandía, Ebro, Tyrrhenian, Eastern Basin, and Turkey). The choice of these two groups (representative of native and invaded areas and with comparable sample size) allows avoiding biased results when using all available sequences of *C. sapidus*, provided that the generated COI sequences for the native range markedly outnumber those obtained so far for the invaded sampling area. Genetic variability was estimated for each population, as well as for each regional dataset, by means of number of haplotypes (*N*h), haplotype (*h*) and nucleotide (*π*) diversities, and the mean number of nucleotide differences (K). These measurements were determined with the software DNASP 5.10 [36] and compared by means of one-way analysis of variance (one way-ANOVA), as implemented in the software PAST 2.17 [37]. For the latter kind of analysis, we intended to seek whether both regional datasets (native and invaded sampling sites, with almost equal sample sizes) could differ statistically in their genetic diversities. The one-way ANOVA is regarded as the most appropriate statistical tool to determine whether the means of the two sample are significantly different or not. Aiming to test the null hypothesis of equal mean values among the tested groups, this statistical approach yields an F-statistic, defined as the ratio of variance calculated among the means to the variance within the samples. A higher ratio allows rejecting the null hypothesis and indicates that the samples were drawn from groups with different mean values. Genetic imprints of historical population dynamics and fluctuations in effective population size were determined through analysis of the two neutrality tests Tajima’s *D* [38] and Fu’s *F*s [39]. These statistics, alongside their level of significance, were estimated with the software Arlequin, using 1000 coalescent simulations. Significantly negative outputs of *D* and *F*s are reliable indicators of demographic expansion [39,40]. Positive values of Tajima’s *D* and Fu’s *F*s, however, usually hint at the occurrence of population demographic contractions or genetic bottlenecks [41]. Provided that bottlenecked populations are expected to be genetically distinct from source populations, exhibiting low levels of genetic diversity, we also intended to seek evidence for significant genetic differentiation between populations of the blue crab *C. sapidus* from native and invaded sampling sites. We used the same statistical procedure, implemented in Arlequin, as previously described to assess pairwise comparisons of genetic differentiation.

## 3. Results

For the construction of three haplotype networks of *Callinectes sapidus* based on different regions of the mitochondrial molecular marker COI, we used all available sequences (self-generated and from GenBank) and listed them in Tables S3–S5 of the Supplementary Material.

**Figure 2:**
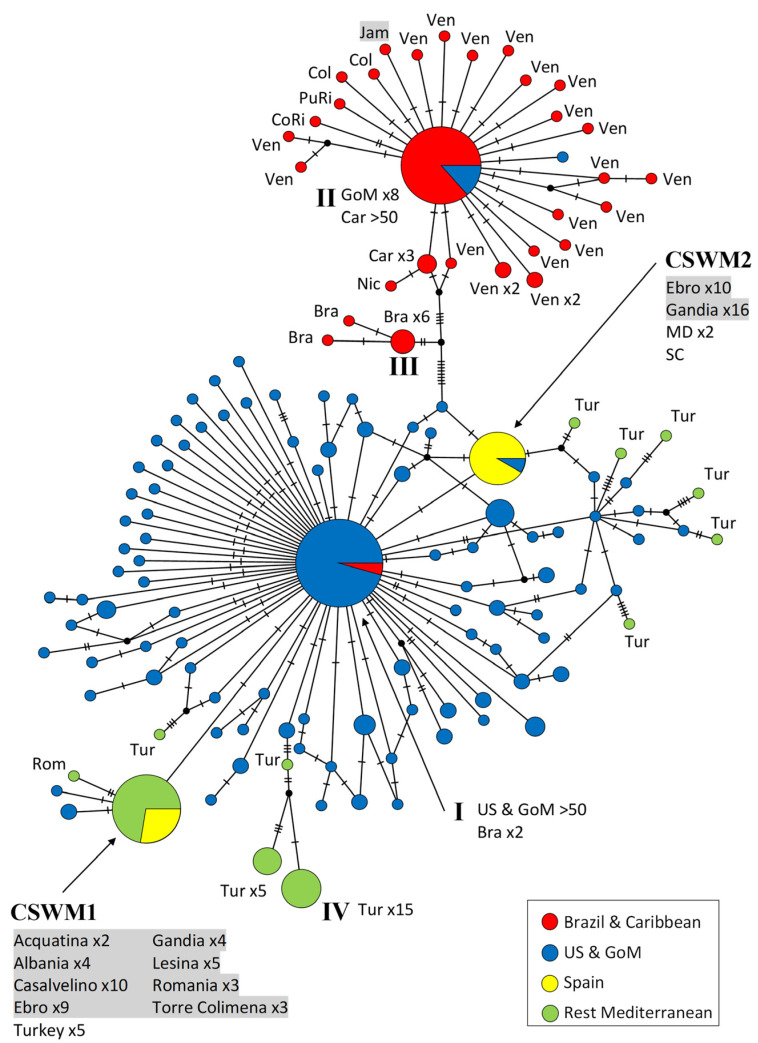
Maximum parsimony network showing genetic distances among 412 sequences of *Callinectes sapidus* corresponding to the ‘Folmer region’ of COI (644 bp; 71 current study, 341 GenBank), constructed with TCS (Popart vers. 1.7). Each circle represents one haplotype. Diameters of the circles correspond to the frequencies of the respective haplotypes. The numbers of dashes display mutational steps (each dash stands for one single nucleotide mutation). Small black circles represent hypothetical (missing) haplotypes. Sample locations are represented by colors, with the North American lineage in blue, South American and Caribbean lineages in red, Spanish ones in yellow, and the remaining Mediterranean Sea in green. Self-generated sequences are shown as grey fields. Abbreviations: Bra = Brazil, Car = Caribbean Sea, Col = Colombia, CoRi = Costa Rica, Jam = Jamaica, GoM = Gulf of Mexico, Mex = Mexico, Nic = Nicaragua, PuRi = Puerto Rico, Rom = Romania, Ven = Venezuela; MD = Maryland, SC = South Carolina. Numbered haplotypes: I = most common North American haplotype; II = most common Caribbean haplotype; III = most common Brazilian haplotype; IV = most common haplotype recorded from Turkey.

The first haplotype network (Figure 2) is based on the 5′ end of the COI gene (Folmer or barcoding region) and designed to include a maximum of blue crab individuals from North, Central, and South America, as well as from Europe. This network comprises 412 sequences with a length of 644 bp. A total of 139 different haplotypes could be detected among the examined individuals, of which 108 haplotypes are singletons (only found in single individuals). There is a noticeable distinction of a haplotype cluster with individuals from South America and the Caribbean, compared to the North American one. In the Caribbean, one very frequent haplotype (ht II) is found in more than 50 individuals. This haplotype differs by at least 14 mutations in its nucleotide composition compared to the North American and European haplotypes. Moreover, the high number of singletons also reflects a high genetic diversity in the Caribbean Sea. The most common haplotype of the Brazilian population (ht III) was detected in six specimens and differs from the Caribbean haplotypes in at least eight nucleotides, and ten from the nearest North American and European haplotypes. The network is dominated by the specimens from the US Atlantic coast and the Gulf of Mexico. The inclusion of the large proportion of sequences from this geographical region in the network analysis is reflected in the occurrence of a very high number of haplotypes: 99 different ones were determined across the sampling sites from the US Atlantic coast and the Gulf of Mexico. One of these haplotypes (ht I) is particularly dominant, as it was detected in more than 50 specimens. Furthermore, this haplotype was also documented in two Brazilian individuals, but has so far not been detected in the Mediterranean Sea. In the European samples, except for Turkey, two other haplotypes were identified as dominant. Compared to each other, these two haplotypes show deviations in two nucleotide bases along the analyzed 644 bp, and the most common North American haplotype is placed in between. One of the haplotypes occurring frequently in Europe (CSWM1 in [30]) was recorded in 45 specimens from Albania, Romania, Italy, and some from Turkey. The second main haplotype from Europe (CSWM2 in [30]) was reported from 29 samples, most of which originate from the eastern Spanish localities Ebro and Gandía. The very same haplotype was also reported from two individuals from Maryland and one from South Carolina. Other haplotypes reported from Turkey appeared in peculiar and unique positions of this network, and the 32 Turkish samples were distributed among 11 different haplotypes. These haplotypes were very conspicuous, due to the separation by at least two nucleotide mutations to the nearest North American or European haplotypes. The most frequent Turkish haplotype (ht IV) was found in fifteen individuals and was isolated from the next North American haplotype by six mutations.

**Figure 3:**
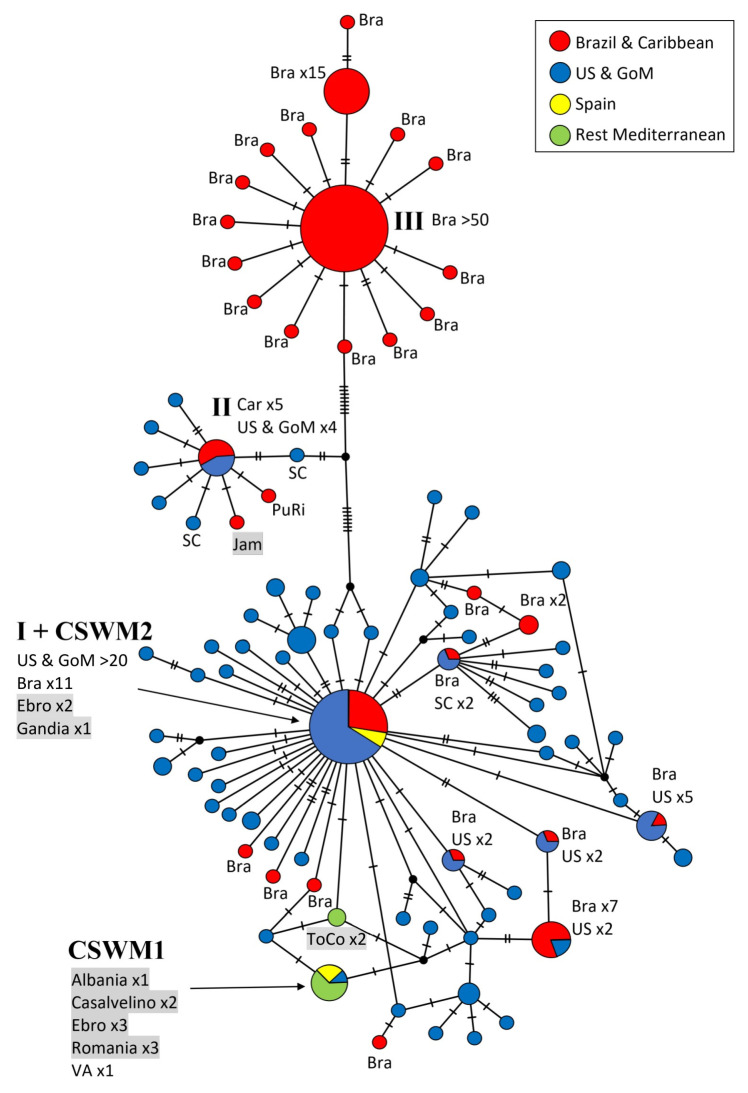
Maximum parsimony network showing genetic distances among 248 sequences of *Callinectes sapidus* corresponding to the ‘Palumbi region’ of COI (640 bp; 16 current study, 232 GenBank), constructed with TCS (Popart vers. 1.7). Diameters of the circles correspond to the frequencies of the respective haplotypes. The numbers of dashes display mutational steps (each dash stands for one single nucleotide mutation). Small black circles represent hypothetical (missing) haplotypes. Sample locations are represented by colors, with the North American lineage in blue, South American and Caribbean lineages in red, Spain in yellow, and the remaining Mediterranean Sea in green. Self-generated sequences are shown as grey fields. Abbreviations: Bra = Brazil, Car = Caribbean Sea, GoM = Gulf of Mexico, Jam = Jamaica, Mex = Mexico, PuRi = Puerto Rico, ToCo = Torre Colimena, Ven = Venezuela; SC = South Carolina, VA = Virginia. Numbered haplotypes: I = most common North American haplotype, including European and Brazilian specimens; II = most common Caribbean haplotype; III = most common Brazilian haplotype.

The second network (Figure 3) is based on 640 bp that are closer to the 3′ end of the COI gene, forming part of the so-called ‘Palumbi region’ and allowing an independent assessment of genetic similarity among blue crab population, as numerous GenBank sequences, especially South American ones, were available exclusively from this gene region, with a minimal overlap of 21 basepairs with the ‘Folmer region’. The network comprises 248 sequences, and the resulting 88 haplotypes are grouped in a similar scheme as in the previous network (Figure 2). In this case, the Caribbean haplotypes (ht II most common one) are clearly separated (up to 12 mutations) from the remaining haplotypes. Seven single haplotypes, mostly originating from Mexico, only differ by one mutation from the main one.

In this network, a closer connection between the Caribbean and North American Atlantic populations seems to be the case. Two single haplotypes from South Carolina were characterized by a great similarity to the central Caribbean haplotype. In this gene region, the highest genetic divergence can be ascribed to Brazilian haplotypes. The central Brazilian haplotype (ht III) was recovered in more than 50 individuals and was markedly distinct from the North American haplotypes (at least 17 fixed mutations). Furthermore, several singletons were present in the Brazilian population, which mostly differ in only one nucleotide base from the main haplotype. This network also demonstrates a high genetic diversity within the distribution area of the US Atlantic coast, including the Gulf of Mexico. This diversity is expressed in the high number of haplotypes, which often show only single or few mutations in their sequences.

The most frequent haplotype (ht I) along the North American Atlantic coast can be considered as the extension of ht I from the ’Folmer region’. However, in this case, this central haplotype was no longer restricted to North American samples, and a few from Brazil, but was also found in three Spanish individuals carrying the western European haplotype (CSWM2). In this DNA region, three different haplotypes were present among the European specimens. The more common and widespread haplotype in Europe (CSWM1) was found in sequences from Ebro, Casalvelino, Romania, Albania, and Virginia. Two specimens from Torre Colimena (ToCo) differ in two positions, forming a separate haplotype. No specimens from Turkey were included in this network, because from there no sequences from the ’Palumbi region’ are available in GenBank.

**Figure 4:**
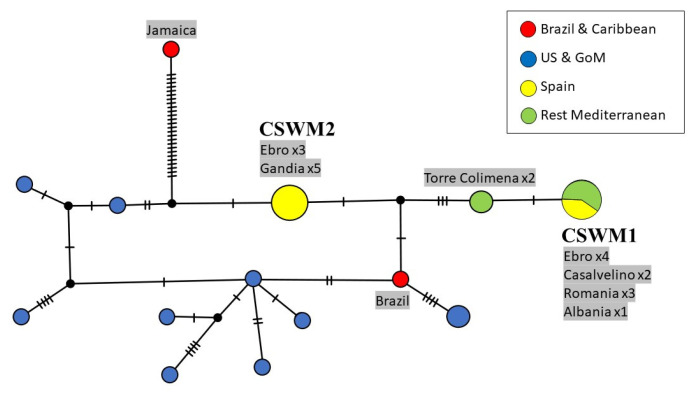
Maximum parsimony network showing genetic distances among 32 sequences of *Callinectes sapidus* combining the ‘Folmer region’ and beginning of the ‘Palumbi region’ of the COI gene (998 bp; 22 current study, 10 GenBank), constructed with TCS (Popart vers. 1.7). Diameters of the circles correspond to the frequencies of the respective haplotypes. The numbers of dashes display mutational steps (each dash stands for one single nucleotide mutation). Small black circles represent hypothetical (missing) haplotypes. Sample locations are represented by colors, with the North American lineage in blue, South American and Caribbean lineages in red, Spain in yellow, and the remaining Mediterranean Sea in green. Self-generated sequences are shown as grey fields. Abbreviations: GoM = Gulf of Mexico.

Genetic differences between American and European populations of *C. sapidus* were also analyzed with a longer COI fragment of 998 bp, as a combination of both previous regions, and are summarized in Figure 4. These 32 sequences encompass the entire ‘Folmer region’ (see Figure 2) and the first half of the ‘Palumbi region’ (see Figure 3), resulting in a total of 17 different haplotypes.

Consistent with the network in Figure 2, the highest nucleotide divergence was found in a Caribbean specimen. In this case, a sequence from Jamaica differs in 27 nucleotide bases from the next Mediterranean haplotype (CSWM2). The only Brazilian individual is nearest to the most common haplotype from the USA and the Gulf of Mexico and separated by two mutations from the next Mediterranean haplotype (CSWM2). However, this specimen also clustered with the North American clade in the previous networks. The number of mutation steps between the two most frequent European haplotypes increased to five.

The one-level AMOVA revealed an overall nucleotide divergence (*Φ*_ST_) of 0.486 and a haplotype frequency (*F*_ST_) of 0.03 (Fixation Index *p* < 0.001; see Table S1 in the Supplementary Material). This significant value indicates the presence of genetic differentiation, and that overall gene flow is restricted. The pairwise comparisons of genetic differentiation are listed in Table 2.

The analysis highlighted significant differences in the haplotype frequencies and nucleotide divergences of the South American population. *Φ*_ST_ values within a range from 0.602 to 0.649 and *F*_ST_ values of 0.054 to 0.075 indicate restricted gene flow to other populations. This genetic differentiation was consistent with the haplotype networks shown in Figure 2 and Figure 4. Moreover, a weakly significant genetic differentiation could be determined between the populations from Maryland and Florida (*p* < 0.05).

The one-level AMOVA of the European populations gives evidence of significant overall genetic differentiation (*Φ*_ST_ = 0.764, *F*_ST_ = 0.492; *p* < 0.001; see Table S2 in Supplementary Material). The pairwise comparison of the included populations showed complete isolation of the Turkish and North American populations (see Table 3).

The individuals from Turkey were genetically differentiated, with highly significant values for both nucleotide divergences and haplotype frequencies (*Φ*_ST_ = 0.766 to 0.91; *F*_ST_ = 0.307 to 0.736). This divergence from the other populations was already visible in the first haplotype network (Figure 2). Values of *Φ*_ST_ (based on nucleotide diversities), ranging from 0.219 to 0.766, and those of *F*_ST_ (based on haplotype frequencies), from 0.258 to 0.464, of the only North American population include (Virginia) showed the same pattern. An exchange of genes with the other populations can thus be ruled out. Furthermore, no gene flow barriers between the populations from the Tyrrhenian Sea and the Eastern Mediterranean Basin could be detected, as well as between the two Spanish populations. In contrast, the genetic differentiation of the Ebro population to the populations from the Tyrrhenian Sea and Eastern Basin revealed weakly significant values (*Φ*_ST_ = 0.416 and 0.476; *F*_ST_ = 0.416 and 0.418, respectively).

The outcome of the genetic diversity analysis based on the examination of 637 basepairs of the mitochondrial COI gene within *C. sapidus* populations is shown in Table 4. Within the native range, the highest level of haplotype diversity (*h*) was recorded in the population of Florida, while those corresponding to the nucleotide diversity (*π*) and mean number of nucleotide differences (K) were highlighted for the South American population. Trends of variation in genetic variability across the invaded sampling area unveiled higher outputs of *h* for the Ebro populations and *π*, and K for the Turkish samples. The lowest levels in these three parameters were recorded in the Tyrrhenian population. Overall, population genetic diversity of the blue crab *C. sapidus* from the native distribution area was found to be markedly higher than that recorded in newly invaded sites for all examined DNA diversity metrics (*h*, *π*, and K) (Table 4). This finding was statistically confirmed by the outcome of the one-way ANOVA (Table 5).

Analyses of the two neutrality tests (Tajima’s *D* and Fu’s *F*s) unraveled contrasting patterns among samples of *C. sapidus* from native and invaded sites, resulting in overall significantly negative outputs within the native range, opposed to positive values of both indices across newly invaded territories (Table 4). These findings point out to disparate trends of historical population dynamics, including past demographic expansion events within the native range and potential signature of recent genetic bottleneck effects within newly established blue crab populations.

The outcome of pairwise comparisons of genetic differentiation within the blue crab *C. sapidus* from native and invaded sites resulted in similar outputs and trends to those already recorded for the two American and European datasets, when analyzed separately. Most importantly, all genetic comparisons involving native and invaded populations were found to be significantly different from each other based on both nucleotide divergence and haplotype frequencies (Table S6).

## 4. Discussion

The phylogeographic comparisons carried out in the present study on native and invasive populations of the Atlantic blue crab *Callinectes sapidus* emphasized the prevalence of three geographically defined lineages among populations from the native range. This was achieved by combining results from two adjacent regions of the COI gene, which so far had been applied independently. One lineage can be assigned to the northwestern Atlantic along the East coast of North America and the Gulf of Mexico (North American lineage), a second one to the Caribbean Sea in Central America (Caribbean lineage), and the third one to the southwest Atlantic (South American lineage). These lineages have already been mentioned in previous studies: Santos & D’Incao [42] and Rodrigues et al. [43] distinguished samples from the northern and southern Atlantic, but without including samples from the Caribbean area, whereas Windsor et al. [44] recognized the Caribbean lineage. The distribution of the blue crab in its native range can thus be subdivided into three main areas that are known to be partly disconnected, for which our study provides further support: new AMOVA results confirm reduced gene flow among these regions and the haplotype networks give evidence for an elevated number of mutation steps among these haplogroups. Various small-scale regional factors could have contributed to this lack of gene flow in the native area, reflecting the phenomenon of repeated isolation in water bodies of different scales [45]. Interestingly, the genetic similarities among the three lineages differ, depending on the analyzed COI gene region, emphasizing the importance of including longer DNA regions or multiple markers, before drawing conclusions on genetic relatedness. Conspicuous is also the fact that several Brazilian specimens of *C. sapidus* (from GenBank and one newly analyzed) cluster within the North American clade, suggesting possible human introductions within the American continent.

In concordance with other sources [46,47,48,49], high genetic diversity was detected in the North American lineage, which can be attributed to a long evolutionary history. Moreover, our networks show that, despite great geographic distances and variation in environmental conditions, there are many shared haplotypes among populations of *C. sapidus* from the Gulf of Mexico and the northwest Atlantic. This low genetic structuring within the North American lineage is supported by the knowledge that a long pelagic larval duration facilitates a high degree of gene flow over long distances and leads to low genetic differentiation, as shown for many other marine species (e.g., [50,51]). In few cases, however, genetic differentiation within *C. sapidus* was detected in locally restricted areas. This includes, for example, the study by Kordos and Burton [52], giving evidence for significant spatial and temporal population genetic differences along the coast from Texas. Moreover, Plough [53] determined low, but significant variation between populations from the north Atlantic and the Gulf of Mexico. A similarly low, but still significant genetic differentiation was also observed in the present study, as the Maryland population had a different genetic structure compared to those from Mexico and Florida. This divergence could be caused by the geographic distance and the currents in the Gulf of Mexico. Circular eddies may increase the regional dispersal potential of the blue crab, as for example demonstrated in phytoplankton [54], but reduce a more distant spread. Another regional geographic pattern proven for *C. sapidus* from the North American lineage is the northward decrease in haplotype diversity along the Atlantic coast [46,55]. The reason for this trend could be temperature fluctuations and post-Pleistocene extension of the distribution range with a bottleneck effect [55]. Along the Brazilian coast, the genetic differentiation of *C. sapidus* has been studied by Lacerda et al. [56]. They discovered high levels of gene flow in blue crab populations over a distance of 740 km along the South American coast. This enormous spreading potential is postulated to be mediated by adult migration as well as larval dispersal, and it may be further facilitated by ship transport and the Brazilian current in the south Atlantic [57].

The ultimate goal of the present study was the genetic characterization of *Callinectes sapidus* in Mediterranean waters, which so far has not been explored at a whole-basin scale. The genetic diversity in the western Mediterranean Basin only reflects a minimal fraction of the one known from the Americas, and the occurrence of only two frequent haplotypes (termed CSWM1 and CSWM2 [30]) can be attributed to a founder effect. The low levels of genetic diversity recorded in examined Mediterranean specimens of *C. sapidus* in comparison to their American counterparts, alongside high genetic differentiation between both population datasets and potential signature of demographic contraction (as evidenced from the analysis of neutrality tests across newly invaded territories), all favor the possibility that the gene pools across invaded sites were shaped by a recent genetic bottleneck, as a consequence of the recent invasion of the Mediterranean Sea. Genetic analyses of complementary nuclear markers in geographically extended and more representative samples from the Mediterranean Sea would help to consolidate these findings. Genetic impoverishment is often found in newly established populations [58]. This effect has also been observed in blue crab populations from New York, which could be attributed to their peripheral location within their range [55]. The low genetic diversity and the restricted gene flow among Mediterranean populations, as well as the similarity to North American populations, suggest that the invasion of the Mediterranean Sea was not a persistent gradual process, but the consequence of a very low number of colonization events, with only few founding individuals. Similar differentiation patterns were also found in the genus *Carcinus*, for which an analysis of microsatellite markers revealed that island populations significantly differ from continental ones [59].

Our results of the pairwise comparisons of genetic differentiation among the European populations are of especially high interest. With a few exceptions, the gene flow among these populations is largely restricted. Significant genetic differentiation could be demonstrated over the entire Mediterranean Sea, as for example between the Spanish and Italian populations or between both and Turkish populations. This genetic structuring stands in sharp contrast with the extensive (mostly unrestricted) gene flow observed in North America. Possibly, several oceanographic factors in the Mediterranean Sea maintain genetic differentiation. This marginal sea is subdivided into several basins characterized by different current systems and ecological conditions [60], and a number of studies have indicated that oceanographic discontinuities, such as currents and eddies, are related to lack of genetic connectivity within different marine species [60,61,62]. The most striking genetic differentiation appears in the blue crab population from Turkey, as indicated by highly significant values in the AMOVA and the presence of many mutation steps in the haplotype networks. The causes for the higher genetic diversity in the Turkish population and the unexpectedly strong differentiation are, on one hand, the longer divergence time, since it is known that *C. sapidus* has been present in the Gulf of Saros and Thessaloniki since 1935 [22,63]. Furthermore, the blue crab was intentionally introduced for aquaculture purposes in those areas [64], and it can be assumed that many founding individuals were used for the initial stocking. However, their strong differentiation from all of the so-far-recorded American haplotypes remains intriguing. Recently, Öztürk et al. [65] genetically characterized *C. sapidus* from the Turkish Black Sea. In agreement with our results, the study showed that the founder of the population encountered in the Black Sea probably originated from the North American lineage. Their comparison of populations from the Black Sea (genetically corresponding to our Romanian specimens) and Levantine Sea (Turkish specimens in GenBank) also suggested a strong differentiation. The physical barriers of the Dardanelles Strait and the Bosporus and the northeastern Aegean Front may maintain restricted gene flow. In general, when determining the impact of hydrodynamic processes on the population genetic connectivity, it is imperative to include seasonality and intensity fluctuations of fronts and currents. This phenomenon is already known from many other fronts and currents [66]; an example from the Mediterranean Sea are the inter-annual changes in the Tyrrhenian flow [67].

An additional potential explanation for the low population-specific connectivity of *C. sapidus* in the Mediterranean Sea could be habitat discontinuities. By virtue of the complex life cycle of the blue crab, temperature and salinity are critical factors in the establishment of populations [68]. The migration of adult blue crab individuals is mainly restricted to estuaries and nearshore coastal waters throughout its native range [24]. After mating in brackish water, females migrate to waters of higher salinity to spawn. The entire duration of the larval development takes place in these waters, and as soon as the megalopa phase is reached, migration back to the original estuaries begins. Optimal conditions for the development of the larvae are 25 °C temperature and a salinity of 30 psu [23]. Deviations from these values could therefore affect the life cycle of this crab species in the Mediterranean Sea, influencing migration behavior and settlement success. An example of the key role of suitable temperatures is the Black Sea: Due to its relatively low temperatures, this area was originally considered hostile to *C. sapidus*. Global warming in recent decades, however, may have facilitated its settlement [22,65].

The larval behavior of *C. sapidus* may represent a further factor driving genetic differentiation in the Mediterranean Sea, as brachyuran larvae can actively choose suitable habitats, e.g., by tidal vertical migration [69], and blue crab megalopae are able to discriminate among habitats, for example between vegetated and non-vegetated areas [70,71]. Accordingly, habitat-related larval and post-larval retention could reduce regional gene flow in *C. sapidus* [52,70,72].

For further research, it will be essential to increase the number of samples in order to supplement the results of our phylogeographic assessment of *C. sapidus* in the Mediterranean Sea. Further sampling in the native region will most likely report (more frequently) the haplotypes predominantly found throughout the Mediterranean Sea. The beneficial effect of including longer DNA regions, as applied in this study, became clear in the haplotype network in Figure 4, when the number of mutation steps recorded between the two western Mediterranean haplotypes increased from two to five, when compared to the classical barcoding region of COI alone (see Figure 2), as a consequence of sequence length extension. This is advantageous for the evaluation at low taxonomic levels, while for the investigation of higher-order phylogenies, more conserved molecular markers should be selected.

To advance our knowledge of the ecology of *Callinectes sapidus* in the Mediterranean Sea, it will be useful to include populations from both sides of potential barriers to gene flow. This way, the influence of hydrodynamic patterns can be directly examined, and other feasible environmental factors can be excluded. Moreover, it would be interesting to draw a comparison among individuals from the Black Sea and the Mediterranean Sea, as only a few samples from Romania could be included in this study. Another factor that should be of importance for further research is the migration behavior of *C. sapidus*. Much is known about migration in the native area of the blue crab, but this knowledge cannot be extrapolated to the Mediterranean Sea without its corresponding verification. It is uncertain whether the life cycle and migration patterns of this species are comparable in the Mediterranean Sea. Since the life cycle is critical to the distribution of this species, future research should focus on it. Analyses of gene flow among Mediterranean populations should be intensified, as this not only reveals information about genetic connectivity, but also enables predictions of the expected speed of further spreading and possible damages caused by this highly invasive species.

## 5. Conclusions

The present study confirms a high level of genetic diversity with extensive gene flow, but three clearly discernible genetic lineages, within the native area of *Callinectes sapidus*. In contrast, a very different population genetic structure becomes apparent in the Mediterranean Sea: Extremely low genetic variability was recorded, probably due to a founder effect, and there is evidence of high genetic differentiation as a consequence of restricted gene flow. The ancestors of the European populations can be traced back to the Atlantic coast of North America. This study represents the first larger-scale assessment of the genetic diversity and reconstruction of the invasion events of *C. sapidus* in the Mediterranean Sea. Since invasive species are known to endanger native biodiversity by unbalancing the stability of ecosystems, and *C. sapidus* is a highly successful invader, further research in this field is essential for considering and developing appropriate conservation measures.

## Figures and Tables

**Table 1:** Collection sites, extraction numbers, coordinates (latitude, longitude), and number of newly analyzed samples of *Callinectes sapidus*.

Sampling Location	Extraction Number	Coordinates	N
Albania: Kavaja	T598	41.18, 19.48	4
Brazil: Ubatuba	L30	−23.44, −45.06	1
Italy: Acquatina	T603	40.44, 18.24	2
Italy: Casalvelino	T675	40.17, 15.12	10
Italy: Lesina	T602	41.89, 15.45	5
Italy: Torre Colimena	T603	40.29, 17.74	3
Jamaica: Belmont	T589	18.16, −78.03	2
Louisiana: Isles Dernieres	L5	29.06, −90.81	1
Mexico: Tampico	T602-7	22.26, −97.78	1
Romania: Constanta	C1-C3	44.18, 28.68	3
Spain: Ebro	T650, T652	40.63, 0.74	19
Spain: Gandía	T649	38.99, −0.15	20
**Total**			**71**

**Table 2:** Pairwise comparisons of genetic differentiation, in American populations of *Callinectes sapidus*, estimated from nucleotide divergence (*Φ*_ST_, below the diagonal) and haplotype frequency (*F*_ST_, above the diagonal). Significant values in bold (*: *p* < 0.05; **: *p* < 0.01; ***: *p* < 0.001) were calculated from 10,000 permutations. Virginia N = 18, Maryland N = 19, Florida N = 13, South Carolina N = 18, Mexico N = 10, South America N = 17.

	Virginia	Maryland	Florida	South Carolina	Mexico	South America
**Virginia**	--	0.025	0.015	−0.008	0.003	0.075 **
**Maryland**	0.021	--	0.024 *	0.003	0.035 *	0.062 **
**Florida**	−0.029	0.047 *	--	0.006	0.01	0.064 **
**South Carolina**	−0.018	0.013	−0.002	--	0.00	0.062 **
**Mexico**	−0.027	0.027	−0.027	−0.007	--	0.054 *
**South America**	0.631 ***	0.649 ***	0.602 ***	0.632 ***	0.607 ***	--

**Table 3:** Pairwise comparisons of genetic differentiation, in European populations of *Callinectes sapidus*, estimated from nucleotide divergence (*Φ*_ST_, below the diagonal) and haplotype frequency (*F*_ST_, above the diagonal). Significant values in bold (*: *p* < 0.05; ***: *p* < 0.001) were calculated from 10,000 permutations. Virginia N = 18, Gandía N = 20, Ebro N = 19, Tyrrhenian N = 10, Eastern Basin N = 18, Turkey N = 18.

	Virginia	Gandía	Ebro	Tyrrhenian	Eastern Basin	Turkey
**Virginia**	--	0.358 ***	0.258 ***	0.441 ***	0.464 ***	0.307 ***
**Gand** **ía**	0.283 ***	--	0.109	0.731 ***	0.718 ***	0.620 ***
**Ebro**	0.219 ***	0.109	--	0.416 *	0.418 ***	0.523 ***
**Tyrrhenian**	0.450 ***	0.731 ***	0.416 *	--	−0.036	0.736 ***
**Eastern Basin**	0.519 ***	0.763 ***	0.476 ***	−0.036	--	0.732 ***
**Turkey**	0.766 ***	0.867 ***	0.844 ***	0.89 ***	0.91 ***	--

**Table 4:** Genetic diversity and demographic estimates for populations of the blue crab *Callinectes sapidus* from native and invaded sampling sites, based on the analysis of 637 basepairs of the mitochondrial COI gene. Values reported for each population as well as for each regional dataset are: sample size (N), number of haplotypes (*N*h), haplotype diversity (*h*), nucleotide diversity (*π*), mean number of nucleotide differences (K), Tajima’s *D* test, and Fu’s *F_S_* test. Significant values of the analyzed neutrality tests (Tajima’s *D*, and Fu’s *F_S_*) are indicated in bold.

**Population**	**N**	***N*** **h**	***h***	***π***	**K**	**Tajima’s *D***	**Fu’s** ***F*** **_S_**
Native sampling sites							
Virginia	18	15	0.961 ± 0.039	0.0048 ± 0.0007	3.091	**−2.030**	**−11.774**
Maryland	19	14	0.953 ± 0.036	0.0037 ± 0.0004	2.385	**−1.660**	**−10.898**
Florida	12	12	1.000 ± 0.034	0.0053 ± 0.0005	3.393	**−1.723**	**−10.5**
South Carolina	18	16	0.987 ± 0.023	0.0045 ± 0.0005	2.895	**−1.957**	**−15.081**
Mexico	10	9	0.978 ± 0.054	0.0038 ± 0.0005	2.422	−1.044	**−6.487**
South America	17	10	0.875 ± 0.07	0.0147 ± 0.0033	9.382	0.381	0.46
Overall data	94	61	0.979 ± 0.007	0.0112 ± 0.0015	7.162	**−1.628**	**−24.923**
Invaded sampling sites							
Gandía	20	2	0.337 ± 0.110	0.0015 ± 0.0005	1.01	0.52	2.973
Ebro	19	2	0.526 ± 0.040	0.0024 ± 0.0001	1.578	2.282	4.317
Tyrrhenian	10	1	0.00 ± 0.00	0.00 ± 0.00	0.00	0.00	0.00
Eastern Basin	18	2	0.111 ± 0.096	0.0001 ± 0.0001	0.111	−1.164	−0.794
Turkey	18	2	0.425 ± 0.099	0.0033 ± 0.0007	2.124	1.459	5.388
Overall data	85	5	0.666 ± 0.031	0.0078 ± 0.0008	5.026	1.393	9.783

**Table 5:** Statistical comparisons of genetic diversity levels among populations of the blue crab *Callinectes sapidus* from native and invaded sampling sites (as indicated in Table 4) by means of one-way analysis of variance (one-way ANOVA). F: F-statistic defined as the ratio of the variance calculated among the means to the variance within the samples. * Significant difference at *p* < 0.05; *** Significant difference at *p* < 0.001.

Genetic Diversity Indices	Statistical Assessment of Difference (One-Way ANOVA) between Native and Invaded Sampling Sites			
	Mean Squares between Groups	Mean Squares within Groups	F	*p*
Haplotype diversity (*h*)	1.257	0.022	56.12	***
Nucleotide diversity (π)	0.00006	0.00001	5.417	*
Mean nucleotide differences (K)	23.953	4.425	5.413	*

## Data Availability

New COI sequences that revealed unrecorded haplotypes, as well as the common haplotypes are retrievable from GenBank (OQ108525- OQ108530).

## Supplementary Materials

The following supporting information can be downloaded at https://www.mdpi.com/article/10.3390/biology12010035/s1: Table S1: One-level AMOVA, based on nucleotide divergences and haplotype frequencies, testing the overall genetic differentiation among American populations of the blue crab *Callinectes sapidus*. Significant values are shown in bold; Table S2: One-level AMOVA, based on nucleotide divergences and haplotype frequencies, testing the overall genetic differentiation among European populations of the blue crab *Callinectes sapidus*. Significant values are shown in bold; Table S3: Sample ID or GenBank ID, sample location, and number of samples of *Callinectes sapidus* included in the first network (‘Folmer region’) (see Figure 2 in main text); * stands for self-generated sequences; Table S4: Sample ID or GenBank ID, sample location and number of samples of *Callinectes sapidus* included in the second network (‘Palumbi region’) (see Figure 3 in main text); * stands for self-generated sequences; Table S5: Sample ID or GenBank ID, sample location and number of samples of *Callinectes sapidus* included in the third network (partly combined ‘Folmer’ and ‘Palumbi’ regions) (see Figure 4 in main text); * stands for self-generated sequences; Table S6: Pairwise comparisons of genetic differentiation for the blue crab *Callinectes sapidus* from native and invaded sampling sites, estimated from nucleotide divergence (*Φ*_ST_, below the diagonal) and haplotype frequency (*F*_ST,_ above the diagonal).
