# Supplementary material for: Phylogeography of the Atlantic Blue Crab Callinectes sapidus (Brachyura: Portunidae) in the Americas versus the Mediterranean Sea: Determining Origins and Genetic Connectivity of a Large-Scale Invasion
Source: Biology (Basel). 2022 Dec 24;12(1):35. doi: 10.3390/biology12010035 (PMC9854962; doi:10.3390/biology12010035)
Supplement: Supplementary file 1 [file biology-12-00035-s001.zip › biology-2074040-supplementary.pdf]

Supplementary Material

Table S1. One-level AMOVA, based on nucleotide divergences and haplotype frequencies, testing the overall genetic differentiation among American populations of the blue crab *Callinectes sapidus*. Significant values are shown in bold.

Source of variation	Degree of freedom	Sum of squares	Variance components	Percentage of variation	Fixation Index (*P*)
Nucleotide divergence (Tajima and Nei distance)					
Among populations	5	161.958	1.954	48.63	**Φ_{ST} = 0.486** ($P < 0.001$)
Within populations	88	181.682	2.064	51.37	
Total	93	343.641	4.018		
Haplotype frequency					
Among populations	5	3.561	0.015	3.07	**F_{ST} = 0.030** ($P < 0.001$)
Within populations	88	42.035	0.477	96.93	
Total	93	45.596	0.492		

Table S2. One-level AMOVA, based on nucleotide divergences and haplotype frequencies, testing the overall genetic differentiation among European populations of the blue crab *Callinectes sapidus*. Significant values are shown in bold.

Source of variation	Degree of freedom	Sum of squares	Variance components	Percentage of variation	Fixation Index (*P*)
Nucleotide divergence (Tajima and Nei distance)					
Among populations	5	183.476	2.111	74.66	**Φ_{ST} = 0.764** ($P < 0.001$)
Within populations	97	69.495	0.716	25.34	
Total	102	252.971	2.827		
Haplotype frequency					
Among populations	5	18.700	0.206	46.29	**F_{ST} = 0.492** ($P < 0.001$)
Within populations	97	20.659	0.212	50.71	
Total	102	39.359	0.419		

Table S3. Sample ID or GenBank ID, sample location, and number of samples of *Callinectes sapidus* included in the first network ('Folmer region') (see Figure 2 in main text); * stands for self-generated sequences.

Sample ID / GenBank ID	Sample location	N
T649 1–20 *	Spain: Gandía	20
T650 1-6 *<br>T652 1-13 *	Spain: Ebro	19
T675 1-10 *	Italy: Casalvelino	10
T602 1-3 *<br>T602 5-6 *	Italy: Lesina	5
T603 1-2 *	Italy: Acquatina	2
T603 3-5 *	Italy: Torre Colimena	3
T598 1-4 *	Albania: nr. Kavaja	4
C1-C3 *	Romania: Constanta	3
JQ435813	Romania: Constanta	1
MG462529 - MG462533	Turkey	5
KC789094 - KC789113	Turkey	20
KC311386, KC311387	Turkey	2
JN561325 - JN561332	Turkey	8
KT073233	Maine	1
AY682072	Connecticut	1
MG462279 - MG462285	New Jersey	7
MG462413 - MG462415	Delaware	3
AY682073, MG462272, MG462416, MG462418, MH985894, MH985899, MH985900, MH985906 - MH985916, KR030239 - KR030243	Maryland	23

MH985931, MH985948, MH985956, MH985965, MH985966, MH985968 - MH985981, MG462275, MG462421, MG462422, MG462424	Virginia	23
MG462407 - MG462412, MH985982	North Carolina	7
AY465915, MH985923, MG462339, MG462449, MG462459, MH985861, MH985872, MH985877 - MH985888	South Carolina	19
MG462464 - MG462469	Georgia	6
MG462326, MG462329, MG462325, MG462447, MG462372, AY682074, MG462355, MG462360, MG462362, MG462363, AY682075, MG462302, AY682076, MH985990, MG462290, MG462434, MG462435	Florida	17
AY363392, AY682077	Mississippi	2
L5 *	Louisiana: Isle Dernieres	1
MG462316, MG462317, MG462505, AY682078	Louisiana	4
AY682079, MG462479, MG462480, MG462440, MG462441	Texas	6
MG462557 - MG462626, NC006281	USA: commercial sample	71
MG462392, MG462393, MG462490	Puerto Rico	3
T602-7 *	Mexico: Tampico	1
MG462364 - MG462369, MG462394 - MG462400, MG462406, MG462492, MG462495, MG462500, MH985988, MH985989, MH985991	Mexico	20
MG462525 - MG462528	Nicaragua	4
84CsaCR	Costa Rica	1
MG462546, MG462549, MG462553, MG462554	Colombia	4
T589 9-10 *	Jamaica: Belmont	2
MH985985 - MH985987, MG462627 - MG462696	Venezuela	73
L30 *	Brazil: Ubatuba	1
MH985983, MH985984, JX123453, MG462250 - MG462256	Brazil	10
		412

Table S4. Sample ID or GenBank ID, sample location and number of samples of *Callinectes sapidus* included in the second network ('Palumbi region') (see Figure 3 in main text); * stands for self-generated sequences.

Sample ID / GenBank ID	Sample location	N
T649-4 *	Spain: Gandía	1
T650 2-3 *<br>T652-1-2, 4 *	Spain: Ebro	5
T675-1, 9 *	Italy: Casalvelino	2
T603-3, 5 *	Italy: Torre Colimena	2
C1-C3 *	Romania: Constanta	3
T598-1 *	Albania: nr. Kavaja	1
NC006281	USA	1
AY682072	Connecticut	1
MH062605	New Jersey	1
MH062590 - MH062591	Delaware	2
AY682073, MH062592 - MH062598, MH062613	Maryland	9
MH062599 - MH062604	Virginia	6
MH062588 - MH062589, MH985992, KU987691 - KU987697	North Carolina	10
MH062626 - MH062637, MH062646 - MH062647	South Carolina	14
MH062638 - MH062645	Georgia	8
MH985993, AY682074 - AY682076, MH062608 - MH062612, MH062614 - MH062615, MH062623 - MH062625, MH062659 - MH062660	Florida	16
AY363392, AY682077	Mississippi	2
AY682078, MH985994, MH062517, MH062518, MH062619 - MH062622	Louisiana	8

AY682079, MH062543 - MH062544, MH062617 - MH062618, MH062648 - MH062658	Texas	16
MH985995 - MH985997, MH062584 - MH062587, MH062662 - MH062672	Mexico	18
T589-9 *	Jamaica: Belmont	1
MH062573, MH986002, MH985998, MH985999	Venezuela	4
L30 *	Brazil: Ubatuba	1
MH062454 - MH062455, MH062457 - MH062459, MH986000 - MH986001, KU987698 - KU987804	Brazil	114
MH062661, MH062469	Puerto Rico	2
		248

Table S5. Sample ID or GenBank ID, sample location and number of samples of *Callinectes sapidus* included in the third network (partly combined 'Folmer' and 'Palumbi' regions) (see Figure 4 in main text); * stands for self-generated sequences.

Sample ID / GenBank ID	Sample location	N
T649-1, 4, 6, 8, 10 *	Spain: Gandía	5
T650 2-4 *<br>T652-2, 4, 7, 10 *	Spain: Ebro	7
T675-1, 9 *	Italy: Casalvelino	2
T603-3, 5 *	Italy: Torre Colimena	2
C1-C3 *	Romania: Constanta	3
T598-1 *	Albania: Kavaja	1
AY682072	Connecticut	1
AY682073	Maryland	1
AY682074 - AY682076	Florida	3
AY363392, AY682077	Mississippi	2
AY682078	Louisiana	1
AY682079	Texas	1
NC006281	USA	1
T589-9 *	Jamaica: Belmont	1
L30 *	Brazil: Ubatuba	1

Table S6. Pairwise comparisons of genetic differentiation for the blue crab *Callinectes sapidus* from native and invaded sampling sites, estimated from nucleotide divergence (Φ_{ST}, below the diagonal) and haplotype frequency ($F_{ST,}$ above the diagonal).

	Native sampling sites						Invaded sampling sites				
	**Virginia**	**Maryland**	**Florida**	**South Carolina**	**Mexico**	**South America**	**Gandía**	**Ebro**	**Tyrrhenian**	**Eastern Basin**	**Turkey**
Virginia	--	0.025	0.015	-0.008	-0.002	**0.075*****	**0.358*****	**0.258*****	**0.441*****	**0.464*****	**0.307*****
Maryland	0.021	--	**0.024***	0.003	0.014	**0.062****	**0.330*****	**0.239*****	**0.440*****	**0.461*****	**0.308*****
Florida	-0.029	**0.047***	--	0.006	0.010	**0.064****	**0.371*****	**0.257*****	**0.471*****	**0.498*****	**0.312*****
South Carolina	-0.018	0.013	-0.002	--	-0.004	**0.062****	**0.314*****	**0.222*****	**0.428*****	**0.450*****	**0.294*****
Mexico	-0.030	0.026	-0.029	-0.009	--	0.042	**0.398*****	**0.276*****	**0.511*****	**0.536*****	**0.335*****
South America	**0.634*****	**0.651*****	**0.604*****	**0.634*****	**0.612*****	--	**0.404*****	**0.303*****	**0.492*****	**0.513*****	**0.352*****
Gandía	**0.283*****	**0.372*****	**0.290*****	**0.277*****	**0.380*****	**0.680*****	--	0.109	**0.731*****	**0.718*****	**0.620*****
Ebro	**0.219*****	**0.338*****	**0.212****	**0.236*****	**0.276****	**0.670*****	0.109	--	**0.416***	**0.418*****	**0.523*****
Tyrrhenian	**0.450*****	**0.594*****	**0.462*****	**0.497*****	**0.582*****	**0.674*****	**0.731*****	**0.416***	--	-0.036	**0.736*****
Eastern Basin	**0.519*****	**0.650*****	**0.545*****	**0.563*****	**0.657*****	**0.732*****	**0.763*****	**0.476*****	-0.036	--	**0.732*****
Turkey	**0.766*****	**0.783*****	**0.770*****	**0.774*****	**0.794*****	**0.768*****	**0.867*****	**0.844*****	**0.890*****	**0.910*****	--

Values in bold denote significant genetic distances, calculated from 10,000 permutations. * Significant difference at $P < 0.05$; ** significant difference at $P < 0.01$; *** significant difference at $P < 0.001$.
